# Short- and Mid-Term Outcomes of Early Alcohol Septal Ablation Therapy for Patients with Mildly Symptomatic Hypertrophic Obstructive Cardiomyopathy: A Tertiary Center Experience

**DOI:** 10.3390/jcm13051444

**Published:** 2024-03-01

**Authors:** Veysel Oktay, Sukru Arslan, Muhammed Heja Gecit, Zubeyir Bulat, Mehmet Emin Gokce

**Affiliations:** Department of Cardiology, Institute of Cardiology, Istanbul University Cerrahpasa, 34000 Istanbul, Turkey; sukru.arslan@iuc.edu.tr (S.A.); hejagecit@hotmail.com (M.H.G.); zbyrbulat@gmail.com (Z.B.); mehmet_emin_gkc@hotmail.com (M.E.G.)

**Keywords:** alcohol septal ablation, hypertrophic obstructive cardiomyopathy, septal reduction treatment

## Abstract

**Background:** Left ventricular outflow tract obstruction (LVOTO) impairs survival and diminishes quality of life in patients with hypertrophic obstructive cardiomyopathy (HOCM). In this study, we aimed to investigate the safety and the efficacy of earlier alcohol septal ablation (ASA) in patients with HOCM. **Methods:** A total of 47 patients with mildly symptomatic HOCM (NYHA II) and having poor functional capacity despite maximal tolerated medical therapy were included. **Results:** The mean age of the patients was 55 ± 14, and 57% of the patients were male. All clinical endpoint targets including 30 d mortality (1% vs. 0% *p* < 0.01), 30 d adverse complications (10% vs. 0% *p* < 0.01), 30 d complete heart block resulting in need for permanent pacemaker (10% vs. 4.2% *p* < 0.01), more than moderate residual mitral regurgitation (5% vs. 2.1% *p* < 0.01), repeat procedure rate (10% vs. 4.2% *p* < 0.01), improvement of (NYHA) class (90% vs. 95.7% *p* < 0.01), rest and provoked (LVOT) gradient < 50 mmHg (90% vs. 97.8% *p* < 0.01) were significantly reached. **Conclusions:** In patients with mildly symptomatic HOCM (NYHA II), earlier ASA may be performed as an effective and safe procedure in experienced centers.

## 1. Introduction

Hypertrophic cardiomyopathy (HCM) is the most common hereditary cardiomyopathy with a different clinical heterogeneity; its prevalence has been reported to range from 1:200 to 1:500 in Western countries [1]. Due to the advances in imaging modalities such as cardiac magnetic resonance (CMR), the frequency of patients diagnosed with HCM is increasing in clinical practice. HCM is defined by wall thickness ≥ 15 mm in one or more left ventricular myocardial segments, as measured with any imaging modality, that is not explained by abnormal loading conditions. In patients with a positive genetic test or having a history of first-degree family members diagnosed with HCM, a slightly limited threshold (13–14 mm) may be diagnostic [2]. The distribution of left ventricular hypertrophy (LVH) is generally asymmetric and involves the interventricular septum more than the other segments. It is commonly inherited as an autosomal dominant penetrance. It is the most common reason of sudden death in competitive athletes [3]. Approximately 40% of patients have sarcomeric gene mutations, and the two most frequent pathogenic variations are detected in genes encoding the sarcomeric proteins as *beta myosin heavy chain 7 (MYH7)* and *myosin-binding protein C3 (MYBPC3)* [4]. In the case of some metabolic (i.e., Anderson–Fabry, Gaucher and Pompe disease), infiltrative (i.e., hereditary transthyretin cardiac amyloidosis) and mitochondrial diseases (i.e., MELAS syndrome), other uncommon genes are involved [5]. Other patients with HCM have negative tests for sarcomeric variants, and the molecular basis of HCM has not been elucidated completely. The main pathophysiological findings of HCM are LVH generally involving the basal portion of interventricular septum, microvascular ischemia, myocardial fibrosis and diastolic dysfunction [6]. The morphologic features of HCM are marked disarray of cardiac muscle cells and several abnormal intramural coronary arteries with thickened walls and narrowed lumens.

HCM patients may present on a spectrum ranging from a stable benign clinical profile, without need to recommend a major intervention, to progressive heart failure or sudden cardiac death [7]. The most common complaints are chest pain, palpitation and shortness of breath in HCM patients. The objectives of HCM management are based on improving the patients’ symptoms and preventing major cardiovascular complications, including sudden cardiac death. As a consequence of the unique nature of HCM, referring HCM patients to experienced centers specializing in HCM plays an important role in better clinical outcomes.

Seventy percent of patients with HCM (70%) have a resting or exercise-induced left ventricular outflow tract obstruction (LVOTO) [8]. In patients with HCM, a peak LVOT gradient ≥ 30 mmHg at rest or after the Valsalva maneuver measured by continuous Doppler echocardiography or by catheterization indicates an obstructive physiology [9]. The primary component of LVOTO is systolic anterior motion (SAM) of the mitral valve and intrinsic abnormalities (mitral leaflets elongation, abnormal chordal attachment and papillary muscle displacement) of the mitral apparatus. The presence of LVOTO is a major determinant of symptoms and clinical outcomes in HCM patients [10]. LVOTO is often a dynamic process and can be augmented with physiological or pharmacological interventions such as strenuous exercise, the Valsalva manoeuvre or a sympathomimetic agent. Although medical treatment (non-vasodilating beta-blockers, calcium channel antagonists, disopyramide and cardiac myosin inhibitors) can adequately improve symptoms in many cases, 5–10% of patients with HCM remain refractory to pharmaceutical therapy [11]. Beta blockers and non-dihydropyridine calcium channel blockers are the first-line medical agents according to their negative inotropic effects. Disopyramide can be preferred as an adjunctive drug, but the anticholinergic side effects often limit its use, especially in older patients. Cardiac myosin inhibitors (mavacamten and aficamten) have recently been developed as new therapeutic agents in HOCM patients to prevent or delay the need for invasive treatment, but the lack of long-term efficacy and safety information and the high cost of treatment are the main issues with these methods [12]. Invasive treatment of HOCM to reduce LVOTO is recommended as a Class I indication in patients with a LVOTO ≥50 mmHg and having severe symptoms (NYHA functional class III-IV) in spite of maximally tolerated medical therapy [13]. Surgical septal myectomy and the alcohol septal ablation (ASA) procedure both have the same level of recommendation in the guidelines, and both are performed in order to reduce LVOTO. ASA alleviates symptoms by inducing a limited infarction of the upper interventricular septum, resulting in a decrease in LVOTO. Although ASA has similar outcomes to surgery in terms of gradient reduction, symptomatic improvement and mortality, it is associated with a higher risk of heart block requiring permanent pacemaker implantation and increased residual LVOTO, resulting in repeat procedure rates. The procedural success of ASA mainly depends on eligible patient selection and the experience of the operators and centers where it is performed. Based on comprehensive assessment of each patient, advanced age, co-morbidities increasing the surgical risk, patient preferences and the presence of a right bundle branch block (RBBB) on initial electocardiography (ECG) are the main factors in daily practice favoring ASA instead of surgical myectomy in HOCM patients [14]. It should be kept in mind that recommendations for invasive treatment options restricted to mainly severe symptomatic patients were derived from an earlier era of invasive treatment and incomplete understanding of the pathophysiological outcomes of LVOTO.

It is known that patients with high LVOTO, even if mildly symptomatic, have a higher mortality than those without markedly elevated LVOTO [15]. Waiting a long time to make a decision for septal reduction treatment (SRT) may have a negative impact on long-term outcomes, and earlier invasive interventions may be associated with improved prognosis [16]. According to the American Heart Association (AHA)/American College of Cardiology (ACC) guideline for the diagnosis and treatment of patients with HCM, published in 2020, in patients with HOCM, earlier (NYHA II) surgical myectomy performed at comprehensive HCM centers was recommended as a Class 2b indication in the presence of additional clinical risk factors, including poor functional capacity attributable to LVOTO as documented through treadmill exercise testing, or young adults with very high resting LVOTO (>100 mmHg) [17]. In 2023, the European Society of Cardiology (ESC) published guidelines for the management of cardiomyopathies and stated that SRT may be considered in experienced centers with demonstrable low procedural complication rates in patients with mild symptoms (NYHA II) which are refractory to medical therapy; other factors includes patients with a resting or maximum provoked (exercise or with Valsalva maneuvers) gradient of ≥50 mmHg in the presence of additional clinical risk factors, including moderate-to-severe SAM-related mitral regurgitation, atrial fibrillation (AF) or moderate-to-severe left atrial enlargement as a Class 2b indication [18]. When both guideline recommendations are evaluated together in this patient group, it is seen that the role of ASA in patients with mildly asymptomatic HOCM is scarce, and additional clinical studies are necessary. 

In this study, we aimed to identify the efficacy and safety of earlier ASA procedures among patients with mildly symptomatic HOCM (NYHA II) having poor functional capacity as documented through exercise testing, as well as adults with very high resting or a provocable LVOT gradient (>50 mmHg) despite maximal tolerated medical therapy.

## 2. Methods

### 2.1. Patient Selection

A total of 127 consecutive patients with HOCM were evaluated in the Istanbul University Cerrahpasa, Institute of Cardiology, between October 2019 and February 2023. In total, 47 patients with mildly symptomatic HOCM (NYHA II) having poor functional capacity as documented through exercise testing, and patients with resting or provocable LVOT gradients (>50 mmHg) despite maximal tolerated medical therapy, as well as those refusing to undergo surgical myectomy, were included. Functional capacity in metabolic equivalents (METs) was estimated based on standard nomograms, with age and gender taken into account to determine whether prognostically important impairment of physical fitness was present [19]. The study was approved by the Istanbul University Cerrahpasa Clinical Research Ethics Committee and written informed consent was obtained from all patients. The ethics approval number and online confirmation code are (E-74555795-050.04-882012) and (BSCPTAKYTZ, date of approval: 6 December 2023), respectively. 

The diagnosis of HOCM was made by experienced cardiologists based on clinical, echocardiographic, and/or cardiac magnetic resonance imaging modalities. Patients with the following criteria were excluded: (1) presence of need for concomitant cardiac surgery (e.g., coronary artery bypass surgery, intrinsic abnormalities of mitral apparatus or papillary muscles, mid-ventricular obstruction, etc.); (2) septal thickness ≥ 30 mm or <16 mm; (3) absence of appropriate coronary anatomy; (4) presence of complete left bundle branch block (LBBB) on ECG. All ASA procedures were performed by interventional cardiologists experienced in this procedure. A cardiomyopathy team comprising a cardiologist expert and a heart surgeon specialized in HOCM was involved in the decision-making process for all patients. An experienced operator is defined as a physician with a minimum case volume of ≥10 procedures per year [20].

### 2.2. Interventional Procedure

All procedures were performed via the femoral route. Two arterial accesses and one venous access were obtained. One of the arterial accesses was used to perform septal ablation and to measure the aortic pressure, while the other one was used to detect the LV pressure for the assessment of LVOTO. Before the ablation procedure, a diagnostic coronary angiography was performed to exclude coronary artery disease and to also select a potential target septal artery. The course of septal arteries was visualized using the right anterior oblique (RAO) and postero-anterior (PA) cranial projections, while the left anterior oblique views (LAO) were used to determine whether the septal arteries run along the right or left side of the interventricular septum (the selection of right-sided septal branches increases the risk of heart block). 

Next, either the 6 or 7F Extra Back-Up (EBU) guiding catheter was used to engage the ostium of the left main coronary artery (LMCA), and a pigtail catheter was placed into the left ventricle. Baseline hemodynamic gradients (resting, under Valsalva maneuver and postextrasystolic beats) were measured. Since almost half of the patients developed a transient complete heart block, a transvenous temporary pacemaker via femoral vein was inserted into the right ventricle and kept in situ for 2–4 days. Unfractionated heparin (100 units/kg) was used for anticoagulation during the procedure, and an extra support 180 cm 0.014 guide wire was inserted into the targeted septal artery. A 90° bend was given to the distal 3–4 mm of the guide wire tip to help it engage the septal artery. An over-the-wire (OTW) percutaneous transluminal coronary angiography (PTCA) balloon around 20–30% larger than the septal artery diameter was passed over the guidewire into the proximal part of the septal artery. A short OTW balloon of 8–10 mm in length was preferred. After intravenous morphine administration for analgesia, the OTW balloon was inflated (8–10 atm) and the guide wire pulled out. After the OTW balloon inflation, a reduction in the LVOT gradient detected by continuous invasive monitoring was considered as a good target of septal artery for ablation. 

A contrast echocardiography study was performed to confirm that the targeted part of the septum has contrast enhancement and the other regions of the left and right ventricle (papillary muscles, apex and right ventricular free wall, etc.) were not enhanced through contrast echocardiography. Multiple transthoracic echocardiographic (TTE) projections (parasternal short axis, apical four chamber, parasternal long axis) were taken to ensure that contrast opacification was involved in the contact point of the anterior mitral leaflet to the septum. In patients with poor image quality, a transesophageal echocardiography (TEE) was performed. Following the contrast echocardiography, the angiographic contrast agent was forcefully injected through the OTW balloon lumen. This was necessary for three purposes. First, any collaterals or aberrant coronary arteries communicating with an epicardial coronary artery like the right coronary artery (RCA) were identified. If these were present, then the procedure was not carried out. Secondly, the forceful injection revealed any backflow across the OTW balloon. Thirdly, if the OTW balloon position was unstable, this allowed us to see the slippage of the balloon and to reposit it properly. Under the strict control of fluoroscopic, haemodynamic and electrocardiographic examination, ethanol (96%) was injected slowly for 3 min into the septal artery, followed by a 5 min observation period, then deflated and rapidly pulled out. A 1 mL insulin syringe was used for this purpose. The amount of injected alcohol was determined by the septal thickness (1 mL/10 mm). A final angiography was performed to confirm coronary flow in the left anterior descending (LAD) coronary artery and blockage of the targeted septal artery. Acute procedural success was defined as a ≥50% reduction in the peak resting or provoked LVOT gradient with a final residual resting gradient of <20 mmHg [21]. In patients in whom insufficient gradient reduction was observed after the first septal artery ablation, the presence of another appropriate septal artery was determined by contrast echocardiography and was ablated. 

All patients were observed in the coronary intensive care unit (CICU) for 24–48 h, and cardiac biomarkers such as troponin and creatine kinase were measured every 6 to 8 h to document the peak values. The total peak of creatine phosphokinase (CPK) levels >1300 U/L in the first 24 h was also considered as a supportive criterion for the procedural success [22]. If no adverse complications were observed, the temporary pacemaker was removed, and the patients were followed up for an additional 3 days in the service unit. Before the discharge, a 24 h Holter examination was used to detect any cardiac bradyarrhythmias, and the medications taken by the patients before the procedure were adjusted according to their clinical conditions. 

### 2.3. Definitions and Study End Points

In this study, we aimed to determine the clinical outcomes according to targets for the post-ASA procedure described in the 2020 AHA/ACC guidelines for the diagnosis and treatment of patients with hypertrophic cardiomyopathy: (1) 30-day mortality; (2) 30-day adverse complications (tamponade, LAD dissection, infection, major bleeding); (3) 30-day complete heart block resulting in need for permanent pacemaker; (4) more than moderate residual mitral regurgitation; (5) repeat procedure rate; (6) improvement NYHA class; (7) rest or provoked LVOT gradient < 50 mmHg. Patient follow-up was performed in the first 30 days after the procedure and every six months thereafter.

### 2.4. Statistical Analysis

The Statistical Package for the Social Sciences software (version 23, SPSS Inc., Chicago, IL, USA) was used for all statistical calculations. The Kolmogorov–Smirnov test was used to identify the distribution of variables normally. The independent samples *t*-test was used to compare continuous variables distributed normally, and the Mann–Whitney U test was used to compare continuous variables distributed non-normally. The chi-square test was used to compare categorical data. Non-normally distributed continuous data were expressed as the median (interquartile range [IQR]), whereas normally distributed continuous data were expressed as mean ± SD. For all tests, a *p* value of <0.05 was considered statistically significant. 

## 3. Results

A total of 47 HOCM patients (mean age 55 ± 14, 57% male) participated in this study. Before the ASA procedure, the percentage of patients using maximally tolerated doses of beta-blockers and non-dihydropyridine calcium channel blockers were (37/47) 79% and (10/47) 21%, respectively. The ratio of patients using disopyramide in combination with other drugs was only (4/47) 8%. The median estimated functional capacity was 4 (3–6) metabolic equivalents (METs) for women and 5 (4–6) (METs) for men. Clinical and echocardiographic characteristics at baseline and six-month follow-up of the patients are shown in Table 1. The median follow-up to detect all-cause mortality and repeat procedure rate was 26 months (IQR: 11–49 months). The volume of injected alcohol during ASA was 1.3 ± 0.3 mL. The classification of clinical endpoints according to targets for post-ASA procedure described in the 2020 AHA/ACC guidelines for the diagnosis and treatment of patients with HCM are summarized in Table 2. During the median follow-up of 26 months, no mortality (0%) was observed in any patient.

## 4. Discussion

ASA is a minimally invasive procedure used to induce a controlled infarction within the proximal portion of the septum, and has been widely used for nearly three decades [23]. When performed by skilled operators at high-volume centers, ASA has outcomes similar to surgical myectomy in terms of LVOT gradient reduction and symptom improvement [24]. The majority of HCM patients have a resting or provoked LVOTO, and some studies have suggested that LVOTO is an independent predictor of poor prognosis [25]. Additionally, it has shown that a greater decrease in LVOTO after the procedure is closely related with better prognosis [26]. Currently, there is no clear recommendation regarding the application of the ASA procedure in mildly symptomatic HOCM patients with poor functional capacity attributable to LVOTO as documented through treadmill exercise testing. The principal finding of this study is that earlier ASA for carefully selected patients with mild symptoms (NYHA II) having poor functional capacity attributable to severe LVOTO was an effective and safe procedure, according to targets for invasive septal reduction therapies (SRT) outcomes published in the 2020 AHA/ACC HCM guidelines.

In clinical practice, SRT is required in approximately 5–10% of all HOCM patients and up to 30% of tertiary care patients [27]. Surgical myectomy and ASA are both performed in order to relieve LVOTO, and both have the same guideline recommendation level, although randomized trials comparing the two methods have not been performed due to logistical or ethical concerns. However, several observational studies and meta-analyses have demonstrated the relative safety and efficacy of these procedures. Since ASA was first introduced by Sigwart in 1995, there have been several modifications to the procedure that have led to better results, and ASA has become the primary SRT modality in many centers [28]. The choice of SRT should be tailored according to clinical, echocardiographic and angiographic characteristics of the patients in addition to operator experiences. 

There is a significant knowledge gap in the literature for asymptomatic or mildly symptomatic patients with severe LVOTO. Patients with severe LVOTO are under the risk of progression of LV hypertrophy, impaired diastolic functions and increased pulmonary artery pressure. In the follow-up period of patients after ASA, the severity of mitral regurgitation, left ventricular end-diastolic pressure and pulmonary artery pressure are decreased, contributing to the preventive effect on AF and pulmonary hypertension in patients with severe LVOTO [29]. 

The presence of severe LVOTO may be harmful irrespective of the severity of symptoms. Rowin et al. described the close relationship between LVOTO and the risk of heart failure progression. In this study, they found that while the time from initial evaluation to progressive heart failure development was 1.6%/year in non-obstructive patients, this ratio was 3.2%/year and 7.4%/year in patients having an LVOT gradient at provocation and rest, respectively [30]. A study from Mayo Clinic (n-544; 59 ± 16) reported that patients with severe LVOTO (>64 mmHg) at baseline had a 10-year survival rate of only 53%, and severe LVOTO was found to be an independent predictor of adverse clinical outcomes. Indeed, death or severe symptoms occurred in 68% of these patients within 10 years after the first diagnosis [31]. Recently, the Euro-ASA registry results reported that long-term survival after ASA in mildly symptomatic patients with HCM was similar to the expected survival of an age- and sex-matched general population (*p* = 0.62); moreover, patients who underwent ASA had LVOT gradient reduction and symptomatic relief with a low risk of developing heart failure [32]. Similarly, Li et al. reported that mildly symptomatic patients with HCM treated with ASA had comparable survival outcomes to those medically treated patients with the additional improvement in functional capacity and they also showed that all-cause mortality was independently associated with resting LVOTO during the follow-up [33]. Different from these two studies, our study had a prospective design, and the functional capacity of all patients in this study was objectively confirmed with the treadmill exercise test. In addition, the procedural outcomes and the rate of clinical endpoints were evaluated according to the targets of guidelines associated with ASA.

In our study, the rate of complications and permanent pacemaker implantation after ASA procedure was 0% and 4.2%, respectively. A possible reason why the rate of complications and permanent pacemaker implantation is quite low is that the operators performing the procedure were highly experienced and the amount of injected alcohol during ASA was conservative (1.3 ± 0.3 mL). Moreover, all procedures were performed with myocardial contrast echocardiography (MCE), which can correctly identify the size of the septal vascular area and predict the size of the infarction caused by alcohol infusion. One study reported that the use of MCE was associated with a higher percentage of acute (92% vs. 70%) and mid-term (94% vs. 64%) procedural success rates than patients who did not undergo MCE [34]. Another issue that should be considered for our results in terms of permanent pacemaker implantation that the patients with left bundle branch block (LBBB) on initial ECG were excluded from the study, which may have contributed to the low percentage of permanent pacemaker requirement. Atrio-ventricular (AV) block requiring permanent pacing occurs in approximately 10–15% of patients after ASA, whereas this is more common (~50%) in patients with initial LBBB on ECG [35].

During a median follow-up 26 months after ASA procedure, we did not encounter any deaths in our study. Although the relatively short follow-up period in our study may explain the low mortality rates, two clinical studies supported our results that the long-term survival rate was 98.5% in patients undergoing ASA according to 10-year follow-up results [36,37]. Another meta-analysis included 20 clinical studies with a mean follow-up of 47 months; the rate of cardiovascular mortality was reported as 2.48% in patients with HOCM after ASA [38]. Fernandes et al. reported that the survival rates at 1 and 10 years after ASA were 98% and 81%, respectively, and were also comparable to demographically similar controls [39]. The reason why the mortality rate in our study was lower than in the existing literature may be explained by the fact that the patients included in our study were younger than the patients included in other studies; moreover, these patients were mildly symptomatic, and their structural heart changes were not obviously evident.

It seems that in patients with HCM who have severe LVOTO, undergoing an earlier ASA procedure instead of waiting until the development of severe symptoms is associated with improved outcomes. Our results and limited data from clinical studies may support the notion that earlier ASA in dedicated centers for patients with severe LVOTO having poor functional capacity documented through the exercise test may improve clinical outcomes without increased risk of any complication or adverse effect. 

### Limitations

Our study has some limitations. First, this is a single-center study with a relatively small sample size. Second, our center is a highly experienced hospital specializing in ASA; thus, caution is needed while generalizing the current results for the low-volume centers. Third, data on long-term clinical outcomes are not available. Finally, overall clinical endpoints of patients enrolled in this study were not compared with expected clinical endpoints of an age- and sex-matched patient population followed by medical treatment or surgical septal myectomy. 

## 5. Conclusions

In patients with HOCM, earlier ASA performed at comprehensive centers may be a reasonable solution in the presence of poor functional capacity attributable to LVOTO, as documented through treadmill exercise testing in terms of short- and mid-term clinical outcomes. We believe that as a result of large-scale similar and consistent studies on this subject, the recommendation contents will be changed in future guidelines.

## Figures and Tables

**Table 1:** Clinical and echocardiographic characteristics at baseline and six-month follow-up of patients with HOCM treated with ASA.

	Baseline (*n* = 47)	Follow-Up (*n* = 47)	*p* Value
NYHA class	2 ± 0	1.2 ± 0.5	<0.01
LV gradient at rest, mmHg *	43 (20–140)	14 (0–20)	<0.01
LV gradient after provocation, mmHg *	84 (50–156)	26 (5–35)	<0.01
LV ejection fraction, %	60 ± 8	63 ± 5	0.340
Basal septum thickness, mm *	18 (16–23)	16 (14–18)	<0.01
Left atrium diameter, mm	44 ± 0.3	42 ± 0.2	0.03
Pulmonary artery systolic pressure, mmHg	25 ± 7	14 ± 5	<0.01

* Mann–Whitney U test was used for non-normally distributed variables and expressed by median (minimum-maximum). LV: Left ventricular; NYHA: New York Heart Association.

**Table 2:** Example targets for alcohol septal ablation outcomes according to 2020 ACC/AHA HCM guidelines (†).

Events	ASA (†)	Our Procedure, *n* (%)	*p* Value
30-day mortality	≤1%	0%	<0.01
30-day adverse complications (*)	≤10%	0%	<0.01
30-day complete heart block resulting in the need for permanent pacemaker	≤10%	2 (4.2%)	<0.01
More than moderate residual mitral regurgitation (**)	≤5%	1 (2.1%)	<0.01
Repeat procedure rate (***)	≤10%	2 (4.2%)	<0.01
Improvement NYHA ≥ class (**)	>90%	45 (95.7%)	<0.01
Rest and provoked LVOT gradient <50 mmHg (**)	>90%	46 (97.8%)	<0.01

(*) Advers complications are described as tamponade, LAD dissection, infection, major bleeding. NYHA: New York Heart Association; LAD: Left anterior descending artery; LVOT: Left ventricular outflow tract. (**) The marked endpoints were evaluated at the six-month follow-up of the patients after the ASA. (***) The repeat procedure rate was evaluated at a median of 26 months follow-up.

## Data Availability

The data that support the findings of this study are available from the corresponding author, V.O., upon reasonable request.
